# Association Between Resting-State Functional Connectivity and Reading in Two Writing Systems in Japanese Children With and Without Developmental Dyslexia

**DOI:** 10.1089/brain.2020.0759

**Published:** 2020-08-18

**Authors:** Teruo Hashimoto, Hiroki Higuchi, Akira Uno, Susumu Yokota, Kohei Asano, Yasuyuki Taki, Ryuta Kawashima

**Affiliations:** ^1^ Division of Developmental Cognitive Neuroscience, Institute of Development, Aging and Cancer, Tohoku University, Sendai, Japan.; ^2^ Graduate School of Comprehensive Human Sciences, University of Tsukuba, Tsukuba, Japan.; ^3^ Faculty of Art and Science, Kyushu University, Fukuoka, Japan.; ^4^ Kokoro Research Center, Kyoto University, Kyoto, Japan.; ^5^ Department of Nuclear Medicine and Radiology, Institute of Development, Aging and Cancer, Tohoku University, Sendai, Japan.

**Keywords:** dyslexic children, bilateral reading mechanism, left fusiform gyrus, resting-state functional connectivity

## Abstract

***Introduction:*** Japanese is unique, as it features two distinct writing systems that share the same sound and meaning: syllabic Hiragana and logographic Kanji scripts. Acquired reading difficulties in Hiragana and Kanji have been examined in older patients with brain lesions. However, the precise mechanisms underlying deficits in developmental dyslexia (DD) remain unclear.

***Materials and Methods:*** The neural signatures of Japanese children with DD were examined by using resting-state functional magnetic resonance imaging. We examined 22 dyslexic and 46 typically developing (TD) children, aged 7–14 years.

***Results:*** Reading performance in each writing system was correlated with neural connectivity in TD children. In contrast, in children with DD, weak associations between neural connectivity and reading performance were observed. In TD children, Hiragana-reading fluency was positively correlated with the left fusiform gyrus network. No significant correlations between Hiragana fluency and neural connectivity were observed in children with DD. Correspondingly, there were fewer correlations between Kanji accuracy and strength of reading-related connectivity in children with DD, whereas positive correlations with the bilateral fronto-parietal network and negative correlations with the left fusiform network were found in TD children.

***Discussion:*** These data suggest that positive and negative coupling with neural connectivity is associated with developing Japanese reading skills. Further, different neural connectivity correlations between Hiragana fluency and Kanji accuracy were detected in TD children but less in children with DD.

***Conclusion:*** The two writing systems may exert differential effects and deficits on reading in healthy children and in children with DD, respectively.

**Impact statement:**

This is the first study of the precise neurobiological characteristics of dyslexia in Japanese children. Because the Japanese language uniquely features two writing systems and there is a low prevalence of dyslexia among Japanese children, our results from an examination of this population provided unique insights into the neural bases of dyslexia. Using resting-state functional magnetic resonance imaging, we determined possible networks that may be implicated in the reading deficits present in these and other children who suffer from dyslexia.

## Introduction

Developmental dyslexia (DD) is characterized by difficulties with accurate and/or fluent word recognition in school-aged children without comorbid intellectual disability. A universal neurocognitive mechanism of DD has been proposed (Paulesu et al., 2001; Silani et al., 2005). However, the frequency of reading difficulty varies across languages and writing systems (Goswami, 2002). Chinese, which features a unique writing system, shows both differences (Siok et al., 2004; Tong et al., 2015) and similarities (Goswami et al., 2011; Hu et al., 2010) with English in terms of reading difficulty. Similarly, common and disparate mechanisms in alphabetic languages have been presented (Martin et al., 2016) and the critical effects of orthography on DD have been suggested (Borleffs et al., 2018).

Reading accuracy in English is correlated with phonological processing. However, it is correlated with the morphological processing in Chinese (Ruan et al., 2018). Interestingly, both languages evoke distinct patterns of neural activity in Chinese–English bilinguals in adults and children (Ip et al., 2017; Xu et al., 2017). Neuroimaging and psychological studies in bilinguals across alphabetical languages have revealed different effects of orthographical complexity on reading difficulty (Lallier and Carreiras, 2018). Cross-linguistic similarities and differences in reading have been revealed. However, these include differences in both pronunciation and meaning.

The Japanese language has two writing systems that share the same sounds and meanings. These are used simultaneously in a mixed manner: logographic Kanji script (derived from Chinese characters) and syllabic Kana script (with the two distinct Kana subsystems Hiragana and Katakana). Revealing the mechanism underlying DD in Japanese readers who learn both syllabic and logographic scripts in one language will offer a novel, innovative perspective in language research.

Hiragana is a fundamental writing system and all Japanese words can be communicated with it. Kanji is a more advanced system that is studied after Hiragana is learned. Hiragana allows the easy prediction of word pronunciation from spelling, because there is a one-to-one relationship between character and pronunciation (46 basic characters, 25 combinations for voiced consonants, and 33 combinations for contracted sound), which can be associated with the extremely low prevalence of inaccurate Hiragana reading in Japanese individuals [0.2% (Uno et al., 2009)]. Japanese children with DD who have difficulties with fluency in reading Hiragana read it slowly but accurately. This suggests that Hiragana is a unique syllabic script. After learning Hiragana, Japanese children learn 1006 Kanji characters in elementary school. Some children experience difficulties in reading Kanji without time pressure. In addition to morphological complexity, most Kanji characters can be read in more than two different ways. The appropriate reading is determined by the intraword context (Wydell et al., 1993). There are 2136 common Kanji characters with 4388 ways of reading.

Japanese DD is characterized by non-fluent but accurate Hiragana reading and difficulty in Kanji reading (Uno et al., 2009). A phonological processing strategy may be applied for Hiragana reading but a lexical whole-word reading strategy is used for Kanji (Wydell and Butterworth, 1999). Separate cognitive bases that are phonological processes for Hiragana and morphological processes for Kanji have been shown for typical Japanese literacy acquisition (Inoue et al., 2017). Reduced phonological awareness, lower visual perception, and influent digit naming have been shown in Japanese dyslexic children (Goto et al., 2010; Seki et al., 2008; Wakamiya et al., 2011). In addition, verbal memory has been suggested to be associated with reading difficulties in Japanese children (Stevenson et al., 1982). Vocabulary is related to Kanji reading in typically developing (TD) Japanese children but less so in dyslexic children (Uno et al., 2009). These results suggest complex characteristics and multiple cognitive deficits, including visual, phonological, and semantic/lexical processes in Japanese DD derived from two different scripts. Revealing a dissociation between Hiragana and Kanji in Japanese reading and the neural mechanisms of those deficits in development could further our understanding of DD.

Neuropsychological studies in patients with alexia have suggested that the dissociation in reading skills between Hiragana and Kanji is associated with an interhemispheric mechanism (Iwata, 1984; Sakurai, 2004). Neuroimaging studies in healthy adults have demonstrated that syllabic Hiragana is associated with left dominant visual and phonological processes, whereas logographic Kanji involves a bilateral network, including visual, phonological, and semantic processing (Kawabata Duncan et al., 2014; Nakamura et al., 2005; Thuy et al., 2004). Japanese Hiragana and Kanji can be used to detect reading deficits caused by left dominant phonological and bilateral orthographic/semantic processes. Further, children can show differential neural underpinnings than adults in Japanese (Kita et al., 2013).

Task-based neuroimaging studies for DD, where age-dependent reading performance is confounded with task-related performance differences (Koyama et al., 2011; Schlaggar and McCandliss, 2007), require both age- and reading-level-matched controls. Resting-state (task-free) functional connectivity is advantageous for the examination of neural networks implicated in reading across ages. In Spanish TD children, reading fluency performance correlates with left fronto-temporal and striatum networks, suggesting the involvement of broad functions for reading acquisition (Alcauter et al., 2017). Both elevated and diminished connectivity between reading-related regions (the left inferior frontal gyrus [IFG], the middle temporal gyrus [MTG], the inferior parietal lobule [IPL], and the fusiform gyrus [FG]) and other bilateral regions for phonological-semantic and visual-orthographic processes has been reported in German adolescents with DD compared with TD adolescents (Schurz et al., 2015). In a narrow age range (12 ± 1.4 years), reading fluency performance correlates with connectivity of the left fronto-parietal and bilateral occipital-temporal-parietal areas associated with visual processing in TD Chinese-speaking children and Chinese-speaking children with DD (Zhou et al., 2015). In English-speaking children with DD, the fronto-parietal attention network was found to be reduced compared with TD children (Koyama et al., 2013). In addition, improved word reading is correlated with enhanced connectivity of bilateral fronto-parietal and occipito-temporal networks, including the FG for visual attention in English-speaking children with DD (Horowitz-Kraus et al., 2015; Koyama et al., 2013). These studies highlight that reading-associated resting-state functional connectivity has been examined in children and adolescents who use Chinese, English, Germany, and Spanish. However, reading-associated resting-state functional connectivity has not been investigated in Japanese children who use two writing systems as their first language.

We hypothesized that bilateral visual, phonological, and semantic networks are involved in Kanji reading in Japanese children, in addition to the left dominant visual and phonological networks for Hiragana reading. Those involvements may be reduced in children with DD. This magnetic resonance imaging (MRI) study assessed resting-state functional connectivity with seed regions-of-interest (ROIs) to whole-brain analysis in Japanese children with DD and TD controls. We sought to investigate the neural underpinnings of deficits in Hiragana reading fluency and Kanji accuracy. We compared the correlation between resting-state functional connectivity and performances in relevant tasks between DD and TD children.

## Materials and Methods

### Participants

Data were initially collected from 69 children who complained of reading difficulties and were suspected to have DD. Among these children, 28 were diagnosed with DD based on the text reading fluency criteria described next. Four were excluded for a lack of data related to Kanji accuracy. Two children who were diagnosed with attention-deficit/hyperactivity disorder (ADHD) and scored higher than the cut-off point on the ADHD Rating Scale-IV were excluded (DuPaul et al., 1998). Thus, 22 children (19 boys and three girls) with DD aged 7–14 years (mean age: 10.7 years) participated in this study. Three children were left-handed and 19 children were right-handed, as determined by using the Edinburgh Handedness Inventory.

We recruited 85 TD children, including 49 age-matched and non-verbal intellectual level-matched children, as study controls. Three children were excluded based on head motion, the criteria for which are described next. Thus, 46 TD children (40 boys and 6 girls) were analyzed in this study. Two children were left-handed and 44 were right-handed.

The third and fourth editions of the Japanese version of the Wechsler Intelligence Scale for Children (WISC-III or WISC-IV) were used to measure the participants' intelligence quotient (IQ). The DD and TD groups were matched by performance IQ (PIQ) and/or their Perceptual Reasoning Index score (not full-scale IQ [FSIQ]). The inclusion criteria ensured that all participants scored an FSIQ >70. The FSIQ score was used as a covariate of no interest in neuroimaging data analyses to control for the effect of intelligence, including verbal comprehension, perceptual reasoning, working memory, and processing speed. All children were native Japanese speakers with corrected or normal vision and auditory function. No apparent brain lesions were checked by one of the authors and a radiologist Y.T. with three-dimensional (3D) T1-weighted images of all participants, which ruled out alexia. According to the Declaration of Helsinki, written informed consent was obtained from the parents of each child before MRI scanning. The study was approved by the Ethics Committees of Tohoku University Medical School and the Faculty of Human Sciences of the University of Tsukuba.

### Reading tests

A standardized reading test [Screening Test of Reading and Writing for Japanese Primary School Children, Revised (Uno et al., 2017)] was used in Japanese children to assess reading fluency across Kanji and Hiragana texts. This is a typical screening test for Japanese children with DD according to the diagnostic guidelines for Japanese dyslexia (Inagaki, 2010). In the Kanji and Hiragana texts consisting of 14 sentences, Hiragana captions were provided for Kanji because some children with DD could not read the text. In addition, we used word reading tests from this screening test to measure reading fluency of 28 Hiragana words and accuracy of 126 Kanji words. Hiragana, unlike English, has very regular (nearly one-to-one) letter-to-sound correspondence. Therefore, reading accuracy tends to be very high for Hiragana with a small variance. Indeed, accuracy levels approach a ceiling by the end of the first school year in the majority of alphabetic languages (Seymour et al., 2003). This was our rationale for analyzing fluency, rather than accuracy, for the text and Hiragana. In addition, no response time (fluency measure) was available for some Kanji words because children with DD cannot read many Kanji words. Thus, accuracy was measured for Kanji reading alone. Z-scores (mean = 0, standard deviation [SD] = 1) were calculated by using the mean and SD scores for each test in each grade of Japanese children to reveal clear differences (positive vs. negative values) between the DD and TD groups. This standardization controlled for the effect of age on reading scores. An SD ≤1.5 for the text (including both Kanji and Hiragana) reading time, with negative values indicating slower reading times, was part of the inclusion and exclusion criteria for DD and TD, respectively. For fluency, a stopwatch was used to measure time to read words and texts. Accuracy of Kanji was scored with written words by hand on paper.

### Psychological test for DD

To assess the visual, phonological, and automatized processing in children with DD, we used the Rey–Osterrieth complex figure test (Osterrieth, 1944), copy, immediate and delayed recall, phonological awareness (backward word repetition), and the rapid automatized naming (RAN) test (both tests in Uno et al., 2017). The Standard Comprehension Test of Abstract Words (Haruhara et al., 2002) was used to assess vocabulary and word comprehension by using abstract words. These psychological tests were conducted only in children with DD, because TD children were expected to score within ±1.5 SD in each psychological test.

### Image acquisition

All images were collected by using a 3 T Philips Intera Achieva (Amsterdam, Netherlands) scanner. The 3D, high-resolution, T1-weighted images were collected by using a magnetization-prepared rapid gradient-echo sequence. The parameters were as follows: 240 × 240 × 162 matrix, repetition time (TR) = 6.5 ms, echo time (TE) = 3 ms, inversion time (TI) = 711 ms, field of view (FOV) = 24 cm, 162 slices, 1.0-mm slice thickness (voxel size: 1.0 × 1.0 × 1.0 mm^3^), and scan duration = 8 min and 3 s. Before the MRI scan, we asked children whether they were anxious about undergoing an MRI. Approximately one-fifth of the children said they were anxious and a mock scanning trial was performed for these children by using a simple model of the MRI scanner that replicated the confined space, darkness, and noise of the actual device.

For resting-state functional MRI (fMRI) studies, 34 trans-axial gradient-echo images (64 × 64 matrix, TR = 2000 ms, TE = 30 ms, FOV = 24 cm, 3.75-mm slice thickness, 3.75 × 3.75 × 3.75 mm^3^ voxel size) covering the entire brain were acquired by using an echo-planar sequence. We obtained 160 functional volumes in this scan while participants were at rest (i.e., supine with their eyes open, motionless, awake, and instructed not to think about anything).

### Resting-state functional connectivity data analyses

The MRI data were preprocessed and analyzed by using the Statistical Parametric Mapping (SPM8) software (Wellcome Department of Cognitive Neurology, London, UK). Resting-state functional connectivity (signal synchrony among remote brain areas) was computed by using simple correlations between spontaneous activation levels in multiple brain areas. No initial volumes were discarded, because the MRI scanner automatically discards initial volumes with a nonsteady state. Before preprocessing, we applied the ArtRepair toolbox (https://cibsr.stanford.edu/tools/human-brain-project/artrepair-software.html) implemented in SPM8 to repair spike noise in slices by interpolation from before and after the scans. The Data Processing Assistant for Resting-State fMRI (DPARSF, http://rfmri.org/DPARSF) was used to preprocess the time series volume of each session per participant. This included realignment to the first volume, slice timing correction, co-registration, normalization to the Montreal Neurological Institute (MNI) space using echo planar imaging templates, spatial smoothing (6-mm full-width half-maximum), detrending, and temporal filtering (0.01–0.1 Hz). After spatial smoothing (before detrending), we used the ArtRepair toolbox to detect and repair bad volumes by interpolation. The criteria for the detection of bad volumes were a 1.5% variation in the global signal intensity and excessive scan-to-scan motion of frame-wise displacement (FD) of 0.5 mm, as previously reported (Schurz et al., 2015). In addition, regressing out nuisance covariates was performed with the Friston-24 model, including 6 head-motion parameters, 6 head-motion parameters from the previous time point, and the 12 corresponding squared items. Moreover, white matter and cerebrospinal fluid signals were regressed out to reduce head motion effects by using an anatomical component-based noise-correction method (Behzadi et al., 2007; Muschelli et al., 2014) with SPM *a priori* masks and the top five principal components. The global mean signal was not regressed out. We used a lenient exclusion criterion of mean FD >0.5 mm (Power et al., 2014) to account for the excessive head movement of children. We did not use more stringent exclusion criteria (FD >0.2 mm), because they reduced the sample size (Satterthwaite et al., 2012). However, our criteria were consistent with previous studies (Koyama et al., 2013; Schurz et al., 2015; Skeide et al., 2015). Based on these criteria, we excluded data from three of the 49 participants in the TD group.

The DPARSF was used for resting-state functional connectivity analyses (voxel-wise, a whole-brain seed-to-voxel analysis). Three regions were used as seed regions based on a previous meta-analysis of functional neuroimaging in reading-related brain areas in participants with DD (Richlan et al., 2011). In addition, we included two regions in the right ventral occipito-temporal area (Wu et al., 2012). Three meta-analysis reports using functional neuroimaging for reading assessment included studies with either Chinese or Japanese participants and these regions were consistently reported as requiring more visual processing for a logographic script (Bolger et al., 2005; Tan et al., 2005; Wu et al., 2012). The left IFG (MNI coordinates of −51, 14, 16), the left IPL (−40, −52, 43), the left FG (−40, −42, −26), the right FG (44, −58, −12), and the right inferior occipital gyrus (28, −86, 0) with a 6-mm radius sphere were defined as seed ROIs. We calculated Pearson's correlations between the mean time course of each ROI and the whole brain, and we transformed these into *z* values at the single-participant level.

We performed two-sample *t*-tests (DD vs. TD) with age, sex, handedness, FSIQ, and FD covariates by using SPM8 for simple group comparisons. Next, 2 (DD vs. TD) by 2 (Hiragana fluency vs. Kanji accuracy) analysis of covariance analyses were performed to examine the group differences in correlations between reading performance scores and functional connectivity by using age, sex, handedness, FSIQ, and FD as covariates. We applied a statistical threshold of family-wise error (FWE) of *p* < 0.05 at the cluster level and an uncorrected *p* < 0.001 at the voxel level. Connectivity strength was extracted from the SPM results (a 6-mm sphere centered at peak coordinates), and Spearman's rank correlations between connectivity strength and reading performance were calculated by using SPSS, version 22 (IBM, Armonk, NY). This was also used for psychological data analysis.

## Results

### Demographics

The mean characteristics of the study participants are shown in Table 1. No significant differences were observed between the groups with respect to age (*t*[66] = 0.51, *p* = 0.61, two-sample *t*-test), the male-female ratio (*χ*^2^[1] = 0.004, *p* = 0.95), or handedness (*χ*^2^[1] = 1.88, *p* = 0.17). Children with DD exhibited lower FSIQ than TD children (*t*[66] = 3.37, *p* = 0.0006). However, their PIQ/Perceptual Reasoning Index scores were similar (98 vs. 101, *t*[30] = 0.77, *p* = 0.44, unequal variance). The mean FD values were 0.29 and 0.24 mm for the DD and TD groups, respectively, and were not significantly different (*t*[33] = 1.76, *p* = 0.09).

**Table 1.** Mean Characteristics of Participants

	N (male:female)	Age	FSIQ^*^	VIQ^*^	PIQ	FD
DD	22 (19:3)	10.7	96	95	98	0.29
TD	46 (40:6)	10.5	107	113	101	0.24

^*^ *p* < 0.001.

DD, developmental dyslexia; FD, frame-wise displacement; FSIQ, full scale IQ; PIQ, performance IQ and perceptual reasoning index; TD, typically developing; VIQ, verbal IQ and verbal comprehension index.

The results of the psychological tests applied to the DD group are shown in Table 2. The vocabulary of children with DD was normal but they showed phonological and automatic processing deficits (Z-scores: ≤1.5 SD). The correlations between reading scores and psychological tests in children with DD are shown in Table 3. Hiragana fluency moderately correlated with automatic processing (RAN), whereas Kanji accuracy correlated with vocabulary.

**Table 2.** Standardized Psychological Performance of Dyslexic Children

Function	Test	Mean scores (SD)
Vocabulary	Standard comprehension test of abstract words	0.18 (0.75)
Phonological	Backward repetition of 3 mola word	−2.86 (5.15)
	Backward repetition of 4 mola word	−1.43 (1.65)
Visual	Rey–Osterrieth complex figure copy	−0.88 (0.97)
	Immediate recall	−0.65 (1.04)
	Delayed recall	−1.21 (1.30)
Automatic	Rapid automatized naming	−1.95 (1.66)

SD, standard deviation.

**Table 3.** Rank Correlations (r) Between Reading Scores and Psychological Performance in Dyslexic Children

		Phonological		Visual			
	Vocabulary	3 Mola	4 Mola	Copy	Immediate	Delayed	Automatic
Hiragana	−0.25	0.11	0.06	−0.24	−0.04	0.13	0.32
Kanji	0.55	0.19	0.00	0.15	0.29	0.06	0.05

### Reading performance

**FIG. 1.**
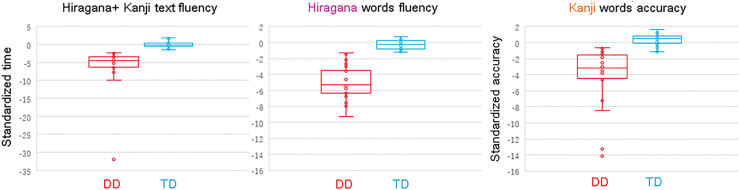
Standardized reading performance in the DD and TD groups. Reading fluency performance in tasks involving Japanese text comprising Kanji and Kana (left panel) or Hiragana (center panel) words, and the reading accuracy of Kanji words (right panel) are shown. DD, developmental dyslexia; TD, typically developing. Color images are available online.

Reading fluency scores in Hiragana and Kanji text, Hiragana word reading fluency scores, and Kanji word reading accuracy scores are shown in Figure 1. The DD group exhibited severe reading difficulties in terms of standardized mean text reading time (−6.07 vs. −0.06, *t*[21] = 4.60, *p* = 0.0001), Hiragana word reading time (−4.98 vs. −0.24, *t*[23] = 10.09, *p* < 0.0001), and Kanji word reading accuracy (−4.10 vs. 0.47, *t*[24] = 5.77, *p* < 0.0001) compared with the TD group. Correlations between reading scores are shown in Table 4. The correlations between Hiragana fluency and Kanji accuracy were low in both groups.

**Table 4.** Rank Correlations Between Reading Scores in Each Group

		Hiragana fluency	Kanji accuracy
DD	Text fluency	0.69	0.50
	Hiragana fluency		0.31
TD	Text fluency	0.59	0.51
	Hiragana fluency		0.22

### Simple group differences in resting-state functional connectivity

No significant differences were observed between TD (*N* = 46) and DD (*N* = 22) groups in resting-state functional connectivity.

### Group by script (Hiragana vs. Kanji) interaction on resting-state functional connectivity

Significant group (TD vs. DD) × script (Hiragana vs. Kanji) interactions were observed in four out of five seed ROI networks (Table 5). Those four ROIs were the left FG (*F*[2,57] = 15.52, *p* = 0.005, FWE corrected at cluster level), the left IPL (*F*[2,57] = 19.48, *p* = 0.004), the left IFG (*F*[2,57] = 11.36, *p* < 0.001), and the right FG (*F*[2,57] = 18.84, *p* = 0.005).

**Table 5.** Connectivity Showing Groups × Scripts Interactions

Connectivity between ROI and brain area				Peak MNI coordinates		
Seed ROIs	Brain areas	Voxels	z Value	x	y	z
Left FG	Left VLPFC	97	4.97	−41	28	−12
	Left MTG	70	4.45	−53	−17	−12
	Right putamen	32	4.02	30	9	−5
	Left IPL	28	3.98	−45	−59	56
Left IPL	Right MFG	44	4.47	53	17	33
Left IFG	Precuneus	47	3.81	−19	−51	59
Right FG	Left PrCG	45	4.89	−41	−14	37

FG, fusiform gyrus; IFG, inferior frontal gyrus; IPL, inferior parietal lobule; MFG, middle frontal gyrus; MNI, Montreal Neurological Institute; MTG, middle temporal gyrus; PrCG, precentral gyrus; ROI, regions-of-interest; VLPFC, ventrolateral prefrontal cortex.

### Group differences in correlations between Hiragana fluency and resting-state functional connectivity

**FIG. 2.**
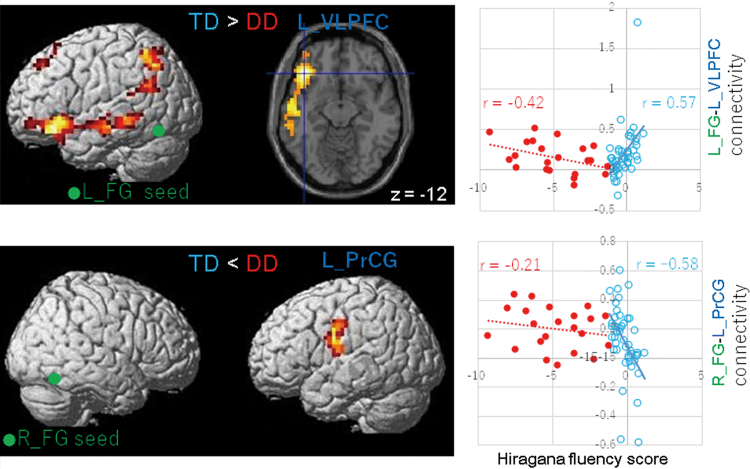
Differences in neural connections correlated with Hiragana reading fluency between DD children and TD children. The seed regions of the left FG (top) and the right FG (bottom) are displayed in green. The strength of connectivity between the left FG seed and the left VLPFC was positively correlated with Hiragana fluency scores in TD children. In contrast, the strength of connectivity between the right FG and the left PrCG was negatively correlated with Hiragana fluency scores in TD children. Reduced correlations were observed in connectivity in children with DD. Lateral views and an axial section with the *z* coordinate are shown. Relationships between Hiragana fluency scores and connectivity between the seed ROI and brain region (dark blue in the left panels) are shown in scatter plots (right panels) with regression lines for each group. Spearman's rank correlation coefficients (*r*) are displayed. The red and sky-blue colors represent the DD and TD groups, respectively. The FWE-corrected *p* < 0.05 was applied at the cluster level, and uncorrected *p* < 0.001 was applied at the voxel level. FWE, family-wise error; L FG, left fusiform gyrus; L PrCG, left precentral gyrus; L VLPFC, left ventrolateral prefrontal cortex; R FG, right fusiform gyrus; ROI, regions-of-interest. Color images are available online.

Connectivity strength analysis between the seed regions and cortical areas showed strong correlations with fluency scores in TD children and fewer correlations in children with DD (Table 6). In TD children, fluent reading was positively correlated with connectivity strength between the left FG and left ventral fronto-temporal areas, including the ventrolateral prefrontal cortex (VLPFC; Fig. 2, upper panel), the MTG, and the anterior temporal lobe (ATL). In addition, the left FG network included the right ATL and the medial prefrontal cortex (MPFC) that positively correlated with Hiragana fluency. Conversely, reading fluency negatively correlated with the connectivity strength between the right FG seed ROIs and the left precentral gyrus (PrCG) in TD children (Fig. 2, bottom panel). In contrast, Hiragana fluency in children with DD showed fewer correlations with those connections than in TD children.

**Table 6.** Connectivity Showing Group Differences in Correlations with Hiragana Fluency

Connectivity between ROI and brain area				Peak MNI coordinates		
Seed ROIs	Brain areas	Voxels	z Value	x	y	z
TD > DD						
Left FG	Left VLPFC	129	5.05	−41	28	−12
	Left temporo-parietal junction	57	4.58	−34	−59	22
	Left IPL	65	4.29	−45	−59	56
	Right ATL	37	4.22	41	9	−35
	Left MTG	82	4.13	−53	−17	−12
	Left ATL	37	4.01	−45	9	−38
	MPFC	49	3.95	−11	39	44
TD < DD						
Right FG	Left PrCG	86	4.23	−41	−14	37

*N* = 46 and 22 for TD and DD, respectively.

ATL, anterior temporal lobe; MPFC, medial prefrontal cortex.

### Group differences in correlations between Kanji accuracy and resting-state functional connectivity

**FIG. 3.**
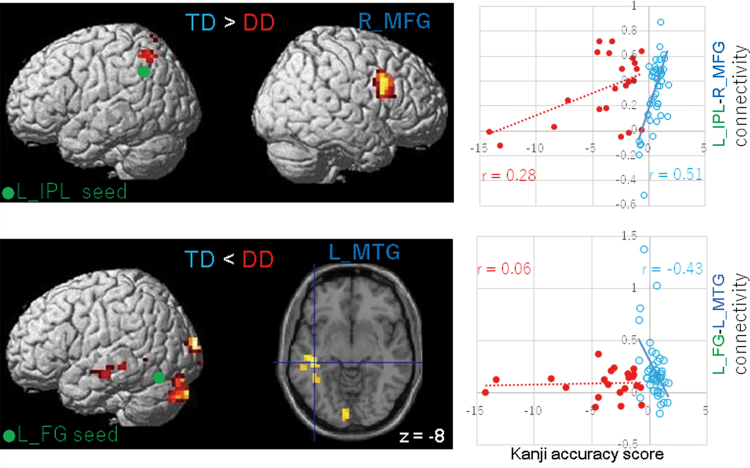
Differences in the neural connections correlated with Kanji reading accuracy between DD children and TD children. The seed regions of the left FG and IPL are displayed in green. Connectivity strength between each seed ROI and brain region (left panel) is positively (upper three panels) or negatively (bottom panel) correlated with Kanji accuracy scores in TD children. Weak correlations were observed in connectivity in children with DD. Lateral views and an axial section with the *z* coordinate are shown. Relationships between Kanji accuracy scores and connectivity between the seed ROI and brain regions (dark blue in the left panels) are shown in scatter plots (right panels), with regression lines for each group. Spearman's correlation rank coefficients (*r*) are shown. The red and sky-clue colors represent the DD and TD groups, respectively. The FWE-corrected *p* < 0.05 was applied at the cluster level, and the uncorrected *p* < 0.001 was applied at the voxel level. L IPL, left inferior parietal lobule; L MTG, left middle temporal gyrus; R MFG, right middle frontal gyrus. Color images are available online.

Kanji accuracy positively correlated with the bilateral network and negatively correlated with the left occipito-temporal network in TD children (Table 7). The strength of connectivity between the left IPL seed ROI and the right middle frontal gyrus (MFG) was more positively correlated with Kanji accuracy scores in TD children than in children with DD (Fig. 3, upper panel). In addition, the strength of connectivity between the left IPL and the superior parietal lobule, between the left IFG and the precuneus, and between the right FG and the left PrCG showed similar relationships. Negative correlations between Kanji accuracy scores and the connectivity strength between the left FG seed ROI and the left MTG were observed in children with TD, whereas no correlations were found in children with DD (Fig. 3, bottom). The connectivity strength between the left FG and the superior occipital gyrus (SOG)/cerebellum/VLPFC also showed negative correlations with Kanji accuracy in TD children.

**Table 7.** Connectivity Showing Group Differences in Correlations with Kanji Accuracy

Connectivity between ROI and brain area				Peak MNI coordinates		
Seed ROIs	Brain areas	Voxels	z Value	x	y	z
TD > DD						
Left IPL	Right MFG	87	4.95	53	17	33
	Left superior parietal lobule	53	4.34	−34	−59	52
Left IFG	Precuneus	115	4.09	−19	−51	59
Right FG	Left PrCG	40	4.55	−44	−14	37
TD < DD						
Left FG	Left SOG	47	4.67	−11	−104	14
	Left cerebellum (Crus I)	104	4.39	−30	−81	−35
	Left MTG	60	4.30	−41	−29	−8
	Left VLPFC	37	3.88	−38	32	−12

*N* = 46 and 22 for TD and DD, respectively.

SOG, superior occipital gyrus.

### Hiragana fluency and Kanji accuracy differences

**FIG. 4.**
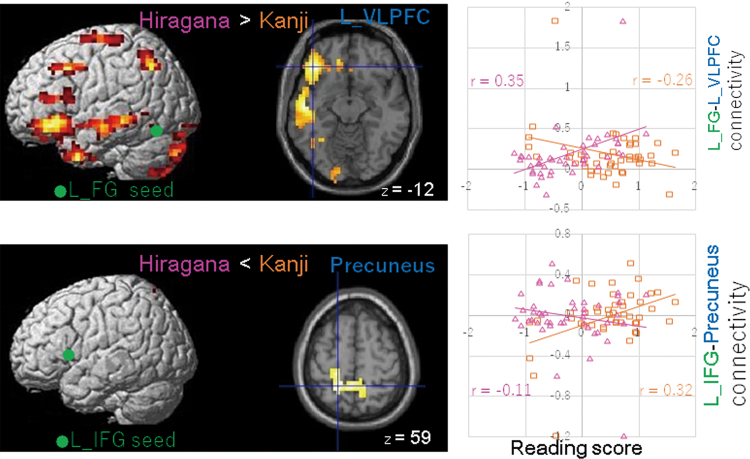
Differences in neural connectivity between Hiragana and Kanji in the TD group. The seed regions of the left FG and the IFG are displayed in green. Connectivity strength between each seed ROI and brain region (left panel) is positively or negatively correlated with Hiragana fluency and Kanji accuracy scores in TD children. Lateral views and an axial section with the *z* coordinate are shown. Relationships between Hiragana and Kanji scores and connectivity between the seed ROI and brain regions (dark blue in the left panels) are shown in scatter plots (right panels), with regression lines for each score. Spearman's rank correlation coefficients (*r*) are shown. The pink and orange colors represent Hiragana and Kanji scores, respectively. The FWE-corrected *p* < 0.05 was applied at the cluster level, and uncorrected *p* < 0.001 was applied at the voxel level. L IFG, left inferior frontal gyrus. Color images are available online.

Differences in neural connectivity between Hiragana and Kanji within the TD group were generally consistent with those between TD children and children with DD (Table 8). In TD children, Hiragana fluency correlated more with the left FG network, including the left VLPFC (Fig. 4, upper panel), the MTG, the PrCG, and the cerebellum than Kanji accuracy (Fig. 4, upper panel). In addition, there were more correlations in the network between the left IFG and the MPFC with Hiragana when compared with Kanji. There were more correlations in the bilateral network between the left IPL and the right MFG, the left IFG and the precuneus (Fig. 4, bottom), and the right FG and the left PrCG in Kanji accuracy when compared with Hiragana fluency.

**Table 8.** Connectivity Showing Differential Correlations Between Kana and Kanji in Each Group

Connectivity between ROI and brain area				Peak MNI coordinates		
Seed ROIs	Brain areas	Voxels	z Value	x	y	z
TD						
Hiragana > Kanji						
Left FG	Left VLPFC	158	5.24	−41	32	−12
	Left MTG	197	5.19	−53	−17	−12
	Right cerebellum (lobule VIII)	186	4.84	23	−70	−53
	Left PrCG	53	4.55	−30	−2	71
	Left cerebellum (Crus I)	37	4.51	−26	−66	−53
	Ventromedial prefrontal cortex	43	4.42	−11	39	−20
	Left ATL	62	4.42	−49	9	−38
	Left MFG	65	4.24	−38	13	41
	Precuneus	41	4.13	11	−36	74
	Right ATL	67	4.12	45	13	−31
	Left IPL	71	3.93	−38	−59	44
Left IFG	MPFC	96	4.43	−19	58	14
Kanji > Hiragana						
Left IPL	Right MFG	66	4.73	49	17	33
	Precuneus	66	3.76	−4	−55	71
Left IFG	Precuneus	93	4.09	−19	−47	59
Right FG	Left PrCG	89	5.62	−41	−14	37
DD						
Hiragana > Kanji	—					
Kanji > Hiragana						
Left IPL	Left SOG	37	4.09	−19	−77	26

*N* = 46 and 22 for TD and DD, respectively.

**FIG. 5.**
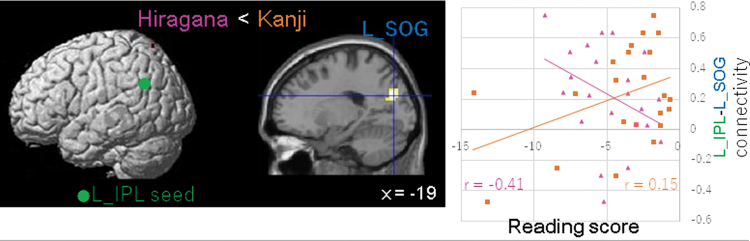
Differences in neural connectivity between Kanji and Hiragana in the DD group. The seed region of the left IPL is displayed in green. Connectivity strength between the seed ROI and the L SOG (left panel) is positively or negatively correlated with Kanji accuracy and Hiragana fluency scores in DD children. Lateral views and a sagittal section with the *x* coordinate are shown. Relationships between Hiragana and Kanji scores and connectivity between the seed ROI and the left SOG are shown in scatter plots (right panel), with regression lines for each score. Spearman's rank correlation coefficients (*r*) are shown. The pink and orange colors represent Hiragana and Kanji scores, respectively. FWE-corrected *p* < 0.05 was applied at the cluster level, and uncorrected *p* < 0.001 was applied at the voxel level. L SOG, left superior occipital gyrus. Color images are available online.

In DD children, Kanji accuracy correlated more with connectivity strength between the left IPL and the SOG than Hiragana fluency (Fig. 5). No further significant differences in correlations were detected between Hiragana and Kanji in children with DD.

## Discussion

To the best of our knowledge, this is the first study to reveal functional connectivity characteristics of Japanese children with and without DD. Reading scores in children with DD exhibited a weak relationship with functional connectivity when compared with TD children, who showed significant positive and negative correlations with connectivity. In other words, less variance in reading performance in TD children could give rise to a stronger correlation with neural connectivity and relatively weaker association between reading and connectivity in children with DD. The left hemispheric and interhemispheric networks for Hiragana and Kanji, respectively, were detected in the TD group. Conversely, we observed less involvement of these networks in the DD group. Moreover, Hiragana fluency and Kanji accuracy showed a differential relationship with functional connectivity in TD children and a few differences in children with DD. These results demonstrate that dissociable mechanisms underlie the ability of children to read Hiragana and Kanji in TD children, and that these mechanisms are not observed in children with DD.

Psychological tests revealed that Hiragana fluency scores were correlated with automatic processing, whereas Kanji accuracy correlated with vocabulary in children with DD. These results suggested a dissociation between Hiragana and Kanji in children with DD. However, a weak correlation between Hiragana fluency and Kanji accuracy might suggest some shared deficits. In TD children, both Hiragana fluency and Kanji accuracy showed similar moderate correlations with text fluency and a very weak correlation between Hiragana fluency and Kanji accuracy. These results suggest a dissociation between Hiragana and Kanji in Japanese reading rather than a dissociation between fluency and accuracy in children.

No significant group differences were found in overall resting-state functional connectivity with controlling age, sex, handedness, intelligence, and head motion. Meanwhile, group differences were observed in relationships between reading scores and reading-related networks.

Higher Hiragana fluency scores were associated with enhanced connectivity in the left FG network, including the ventral fronto-temporal regions in TD children. These associations were reduced in children with DD. The left FG network was positively and negatively associated with Hiragana and Kanji, respectively, in TD children. The visual word form area in the left FG is a key region for reading development and expertise (Centanni et al., 2019; Dehaene-Lambertz et al., 2018). Further, a previous study has demonstrated reading-related co-activation and structural connectivity between the left FG and ventral fronto-temporal regions (Bouhali et al., 2014). Expert and automatic visual processing in the left FG and phonological-semantic processing in the left frontotemporal region may be involved in Hiragana fluency. In contrast, the learning of Kanji continues throughout adulthood. Children usually do not exhibit expertise in Kanji, which may explain the reduced associations with the left FG network. The left FG network has shown more involvement in reading with development (Koyama et al., 2011; Moulton et al., 2019). The left FG network may be involved in the reading of more than 2000 Kanji characters after maturity.

Interestingly, the network between the right FG and the left PrCG negatively correlated with Hiragana fluency whereas that network showed greater correlation with Kanji than that with Hiragana in TD children. The right ventral occipito-temporal region is associated with the reading of logographic scripts (Bolger et al., 2005; Tan et al., 2005; Wu et al., 2012). In addition, it is associated with visual orthographic and lexical-semantic processing (Kawabata Duncan et al., 2014; Thuy et al., 2004). A previous study has reported that enhanced right FG activity during a reading task is associated with reading gain in English-speaking children who struggle with reading (Nugiel et al., 2019). In addition, the left PrCG is involved in handwriting (Longcamp et al., 2019; Roux et al., 2009) and Japanese children learn to read and write Kanji by intensive handwriting repetition (Naka and Naoi, 1995). These results suggest that the right FG network might be related to visually demanding Kanji reading and less involved in the phonological and automatic processing required for Hiragana in TD children. This indicates that the functional lateralization of the FG network might be associated with reading development in Japanese TD children, and that those deficits might be involved in reading difficulties in Japanese children with DD.

Higher Kanji reading accuracy was related to greater functional connectivity between the left IPL and right MFG in the TD group compared with the DD group. Dyslexia-related anomalies in the left temporo-parietal region, including the IPL, have been consistently detected in young children (Vandermosten et al., 2016). Further, reduced activity in the left IPL in children with DD is associated with both orthographic and phonological processes (Paz-Alonso et al., 2018). Kanji reading is associated with visual and semantic processes in the right hemisphere (Kawabata Duncan et al., 2014; Nakamura et al., 2005). In addition, the right MFG is associated with phonological and semantic processes for Chinese reading (Wu et al., 2012). This suggests that visual, phonological, and semantic processes may be involved in Kanji reading in Japanese children. In addition, Kanji performance showed a stronger correlation with connectivity between the left IFG/IPL and the precuneus than Hiragana in TD. The left IFG is associated with the ability to access a phonological output representation in adults with DD (Boets et al., 2013). The network of the left IFG and the precuneus may be involved in the naive phonetic processing of Kanji (Williams et al., 2015). Further, bilateral frontal involvement with weak semantic language lateralization is associated with a higher vocabulary in healthy children (Bartha-Doering et al., 2018). The fronto-parietal top-down control network might support rapid adjustment of multiple processes of Kanji reading (Dosenbach et al., 2008). Taken together, we hypothesize that visual, phonological, and semantic/lexical processes in the bilateral fronto-parietal network are required for Kanji (Chinese characters) reading (Pan et al., 2016; Ziegler and Goswami, 2005) and deficits in these networks are associated with reading difficulty in children with DD.

In the DD group, Kanji correlated more with the connectivity between the left IPL and the SOG than Hiragana. A positive correlation in Kanji was derived from reduced connectivity in dyslexic children with severe Kanji reading difficulty, whereas enhanced connectivity with severe Hiragana reading difficulty was observed. The left parieto-occipital network is associated with orthographic and visuo-spatial processes, including working memory (Richter et al., 2019). Enhanced left occipito-parietal activity in TD children and less activity in dyslexic children for orthographic tasks have been reported (Temple et al., 2001). Reduced connectivity in severe Kanji impairment and compensation or erroneous recruiting in influent Hiragana in the left parieto-occipital network might be associated with deficits in visuo-spatial word processing in children with DD.

Several limitations of this study should be mentioned. First, the age range of participants was relatively large and resting-state functional connectivity changes with development (Mak et al., 2017). Reading skills progressively improve at this age. Therefore, how age affects the observed differences in the neural network should be examined in future studies. In addition, our results might reflect fluency/accuracy dissociation rather than actual differences between Hiragana and Kanji. Reading fluency has been associated with processing speed and automatic processing, whereas accuracy has been shown to involve phonological and morphological processing (Shany and Share, 2011). Nevertheless, in addition to differences, similarity and interactions between reading fluency and accuracy in Chinese children have been reported (Zhao et al., 2019). Further, the correlations between reading accuracy and fluency observed in both children with DD and TD suggest some overlapping processes for Kanji and Hiragana reading, and one or both may have different degrees of impairment in Japanese children with DD. Future studies require the enrolment of larger numbers of children with DD. We also note that head motion artifacts may have contaminated our results due to the liberal exclusion criterion used. The short scan length of this study could compromise the reliability of resting-state functional connectivity data (Birn et al., 2013), and future studies with longer scan length are necessary for children with DD. Although we used a standard MNI template for participants aged 7–14 years as previous studies (Alcauter et al., 2017; Koyama et al., 2013; Zhou et al., 2015), using an age-appropriate template could result in better spatial normalization (Sanchez et al., 2012; Wilke et al., 2017). Finally, because of the uniqueness of the Japanese writing system, the relevance of these results to other languages should be analyzed and validated.

## Conclusions

This study revealed distinct neural characteristics of two writing systems in Japanese children with DD, as measured by resting-state functional connectivity. Connectivity enhancements between the left FG and the fronto-temporal circuits may be involved in phonological control for syllabic Hiragana reading. In addition, greater connectivity between the left inferior parietal and the right middle frontal areas may reflect visual, phonological, and semantic processes for logographic Kanji reading. Collectively, these results indicate potential neural mechanisms underlying the dysfunction observed in this unique population of Japanese children with DD.

## Authors' Contributions

T.H. performed MRI data collection of all participants, psychological data collection of TD children, all analyses, and wrote the article. H.H. conducted psychological data collection and analyses of children with DD. A.U. recruited and diagnosed children with DD, and supervised data collection and analyses. S.Y. collected MRI data of all participants. K.A. collected MRI data of children with DD. Y.T. conceived the study and checked structural MRI data. R.K. organized and supervised the study; all authors reviewed and approved the final article.
